# Prevalence and Predictors of Obesity and Overweight among Adults Visiting Primary Care Settings in the Southwestern Region, Saudi Arabia

**DOI:** 10.1155/2019/8073057

**Published:** 2019-03-05

**Authors:** Awad Mohammed Al-Qahtani

**Affiliations:** Department of Family and Community Medicine, College of Medicine, Najran University, Najran, Saudi Arabia

## Abstract

**Introduction:**

Obesity is becoming the most common health problem of the 21st century, as it will contribute significantly to the high prevalence of cardiovascular disease in developing countries. The main objective of our study was to estimate the prevalence of obesity and overweight among adults attending primary health care settings, southwestern region of the Kingdom of Saudi Arabia.

**Methods:**

The studied population was composed of adults visiting primary health care centres in the southwestern region of Saudi Arabia. A cross-sectional study was carried out on a representative sample of 1681 adult patients. Obesity and overweight were defined according to the WHO standards. Statistical analysis was conducted using the statistical package SPSS 17.0. Logistic regression analysis was used to identify independent predictors of obesity and overweight in the studied population.

**Results:**

Data on body mass index (BMI) measurement was recorded for 1649 out of 1681 participants (98.1%). The overall mean weight was 74.1 ± 15.81 kg; and that for men was 77.69 ± 16.14 kg vs. 69.37 ± 14.02 kg for women with significant statistical difference of p < 0.001. The overall prevalence of overweight and obesity was, respectively, 38.3% and 27.6%. Smoking was not significantly associated with obesity, whereas hypertension was significantly associated with obesity. The risk of overweight or obesity significantly increased from the highest to the lowest monthly income; it passed from 1.67 CI 95% = [1.24-2.25] within the category 5000-7000 SAR to 2.23 CI 95% = [1.71-2.90] within the category less than 5000 SAR.

**Conclusion:**

Our study showed high prevalence of overweight and obesity which should be considered as a public health concern to be followed by specific interventions at the community level with multidisciplinary activities starting from childhood as a primordial prevention program.

## 1. Introduction

The global status of obesity has shown an epidemic trend in many developed countries. The same phenomenon is now being observed in developing countries [1–6]. Changes in the world food system have involved developing countries in a nutritional transition characterized by westernized diet and an increasingly sedentary lifestyle [7]. Because of the accelerated nutritional transition and globalization, it is now noted that in developing countries obesity and overweight are found in both poor and rich population [8–11]. Socioeconomic factors influence the occurrence of overweight and obesity in a given population. Recent studies have shown that, in countries with a western-type lifestyle, economically weaker sections and socially disadvantaged groups are more often affected by obesity than are relatively well-to-do individuals. This trend has been recently noticed in Saudi Arabia due to lifestyle transition from traditional Saudi lifestyle towards more western lifestyle. Recent results of the health surveys show obesity is more common in children and adults of low socioeconomic status [12, 13]. Information about education, occupational status, and net household income is used to measure the socioeconomic status; the same methodology was followed in our study to reveal socioeconomic differences affecting the occurrence and prevalence of overweight and obesity [14]. Thus, obesity has become one of the most common health problems of the 21st century, as it will contribute significantly to the high prevalence of cardiovascular disease in developing countries [15]. Obesity is a serious health issue and predisposes individuals to an increased risk of morbidity and mortality from conditions such as diabetes and hypertension [16, 17].

In fact, over the last 20 years, the rates of obesity have tripled largely due to decreased physical activity and increased consumption of energy dense foods [7]. Strong evidence has been reported concerning the increase of heart failure and mortality incidence in relation to adiposity and obesity [16, 17]. Weight management for overweight youth has been associated with significant health benefits, increased physical fitness, and psychosocial effects such as reduced risk for the development of eating disorders [18].

In the southwestern region of the Kingdom of Saudi Arabia, there is scarcity of data about the prevalence or incidence of obesity and overweight at the community level. A diverse set of patients across all age groups and socioeconomic background, with different diseases and conditions, along with the healthy ones visit primary care centres in Saudi Arabia for the purpose of preliminary and primary care. Hence, all the participants were carefully screened for the overweight and obesity. The main objective of this study was to estimate the prevalence and risk factors of obesity and overweight among adults in primary health care settings in southwestern region of Saudi Arabia.

## 2. Patients and Methods

The studied population was composed of adults visiting primary health care centres in the southwestern region of Saudi Arabia. A cross-sectional study was carried out on a representative sample of adults. The sample size calculation was based upon the estimation of an unknown prevalence (p=50%) with a precision of ± 2.5% and a confidence level of 95%. The needed sample size was at least 1600 subjects. All participants completed a self-administered and pretested questionnaire.

Qualified personnel recorded body weight to the nearest 0.1 kg using a standard beam balance scale with subjects barefoot and wearing light indoor clothing. Body height was recorded to the nearest 0.5 cm. Body mass index (BMI) was defined as the ratio of body weight to body height squared, expressed as kg/m^2^. The following definitions were used for the variables of the study:

(i) Obesity and overweight were defined according to the WHO standards. Overweight was defined as BMI equal to or greater than 25. Obesity was defined as BMI equal to or greater than 30.

(ii) Blood pressure: Taking into account risk of bias due to observation, the electronic system was used to measure blood pressure. The reproducibility of measures and the precision of this device have been demonstrated. After 10 minutes of rest, blood pressure (BP) was measured on the right arm in a sitting position, using an appropriate cuff size. The blood pressure was measured again after a 15-minute rest and the average was used in the latest analysis.

High blood pressure was defined according to the latest guidelines of the Joint National Committee (JNC 8). A systolic blood pressure of 140 mmHg or higher was considered to be hypertension, or high blood pressure. A diastolic blood pressure of 90 mmHg or higher was considered to be hypertension or high blood pressure.

(iii) Smoking status: Smoking status was established in accordance to the WHO criteria; the subjects were considered to be smokers if they smoked either daily (at least 1 cigarette per day) or occasionally (less than 1 cigarette per day) at the time of the study. The subjects who smoked previously and had quit smoking were considered to be ex-smokers.

This investigation was undertaken with caution and with respect of the rights and integrity of the subjects. An ethical approval was obtained from the Scientific Committee of Research, College of Medicine, Najran University. All the procedures of the study were fully explained to the potential participants, and they were assured that participation was voluntary and that they could refuse or quit at any time from study without their medical care being affected.

### 2.1. Data Analysis

Statistical analysis was conducted using the statistical package SPSS 17.0. The data were analyzed using the Student* t*-test and chi-square test to compare, respectively, means and percentages between independent groups. Logistic regression analysis was used to identify independent predictors of obesity and overweight in the studied population.

## 3. Results

The present study included 1681 adults (949 males and 732 females). The majority of the studied population was Saudi (79.2%). More than half of them were married (57%) and were educated up to primary level (52.2%) (Table 1). Data on BMI measurement were recorded for 1649 out of 1681 participants (98.1%). The overall mean weight was 74.1 ± 15.81 kg and that for men was 77.69 ± 16.14 kg vs. 69.37 ± 14.02 kg for women with significant statistical difference of p < 0.001. The overall mean height was 1.65 ± 0.09 meters, and that for men was 1.69 + 0.08 meters vs. 1.59 ± 0.08 meters for women with significant statistical difference of p < 0.001. The mean BMI was 27.27 ± 5.58 kg/m^2^. The overall prevalence of overweight and obesity was, respectively, 38.3% and 27.6% (Table 2).  Table 3 presents the distribution of BMI according to the main characteristics of the studied population. The married individuals had the highest BMI, while the unmarried had the lowest (p < 0.001). Logistic regression analysis revealed monthly income to be a significant predictor of overweight or obesity in the studied population. The risk of overweight or obesity significantly increased from the highest to the lowest monthly income. It passed from 1.67 CI 95% = [1.24-2.25] within the category of 5000-7000 SAR to 2.23 CI 95% = [1.71-2.90] within the category of less than 5000 SAR. Hypertension was also significantly associated with obesity. The risk of obesity increased significantly from patients with normal blood pressure to patients with hypertension: OR = 1.65; CI 95%= [1.22-2.224]; p=0.001. Smoking habit was not significantly associated with obesity. The risk of obesity decreased nonsignificantly from nonsmoker patients to smoker patients: OR= 0.82; CI 95%= [0.66-1.02]; p=0.07.

## 4. Discussion

Our study included 1681 adults visiting primary care centres in the southwestern region of Saudi Arabia. The main findings showed that the overall prevalence of overweight and obesity was, respectively, 38.3% and 27.6%. According to gender, obesity was found in 26.2% among men and 29.1% among women. Similar findings were observed at the national level in Saudi Arabia in 2014 according to a national study [19]. This national study showed a female predominance regarding obesity prevalence (33.5% among females vs. 24.1% among males). A similar picture was also noted in some Gulf countries like Kuwait and Qatar. In Kuwait, the overall prevalence of obesity was 42.8%, and in Qatar, 33.1% of the population was considered obese [20].

The high prevalence of obesity in Gulf countries, especially in Saudi Arabia, could be explained in part by the rapid urbanization and the huge lifestyle transformation at the community level characterized especially by a sedentary lifestyle and non-healthy eating habits [7]. In our study, sedentary lifestyle was reported by more than 45% of the studied population. This high level of sedentary lifestyle could be explained especially among women by the restricted access to sport activities in addition to the overuse of internet devices and social media, smart phones, and watching TV programs [21, 22]. It was reported also in previous studies [19, 23] that multiple pregnancies could be considered as a specific female risk factor for obesity which was associated with gestational weight gain and an increase of food intake. The cultural beliefs among women especially after delivery that for at least 40 days women should not practice any kind of physical activities and they should follow high caloric intake regimen with excess of food intake could also be a factor.

Our study showed also a negative association between overweight and obesity levels, with monthly income. Indeed, when the monthly income increased and exceeded 7000 SAR, the prevalence of obesity decreased passing from 34.1% among people with monthly income less than 5000 SAR to 24.1% among people with a monthly income more than 7000 SAR. This finding could be explained partially by the limited access to healthy foods and sport facilities especially for women. The relationship between smoking and obesity is poorly understood. Some previous studies have revealed no significant association between smoking status and BMI [24], whereas other studies have shown that smoking may be associated with lower BMI [25] and ex-smokers were associated with increased BMI [26]. In our study, the occurrence of obesity was less in smokers (30.31%) as compared to nonsmokers (31.94%). Thus, smoking was not significantly associated with obesity and risk of obesity decreased nonsignificantly from nonsmokers to smokers. Obesity is a major risk factor for diabetes and hypertension [27]. In our study, hypertension was significantly associated with obesity. However, hypertension cannot be considered as a predictor of obesity but it is a consequence and that is why it was significantly associated with obesity.

## 5. Conclusion

In conclusion, our study showed high prevalence of overweight and obesity which should be considered as a public health concern and should be followed by specific interventions at the community level, especially targeting the low income citizens, with multidisciplinary activities, starting from childhood as a primordial prevention program.

## Figures and Tables

**Table 1:** General characteristics of the studied population.

Variable	N	%
*Gender*		

Male	949	56.5

Female	732	43.5

*Nationality*		

Saudi	1238	79.2

Non Saudi	326	20.8

*Marital Status*		

Married	946	57.0

Single	600	36.2

Divorced	113	6.8

*Level of education*		

Primary	861	52.2

Secondary	443	26.9

University	344	20.9

*Monthly income*		

>7000 SAR	480	33.4

5000-7000 SAR	289	20.1

<5000 SAR	667	46.4

**Table 2:** General habits and comorbidities of the studied population.

Variable	N	%
*Physical activity practice At least 30 min/day during 5 days/week*		

Yes	898	53.7

No	773	46.3

*Frequency of buying food from restaurant*		

1-3	967	61.1

>3	488	30.9

*History of Diabetes*		

Yes	317	18.9

No	1363	81.1

*History of Hypertension*		

Yes	255	15.2

No	1422	84.8

*History of high cholesterol*		

Yes	283	17

No	1385	83

*Overweight and Obesity*		

Normal	562	34.1

Overweight	632	38.3

Obesity	455	27.6

*Smoking Status*		

Non-Smoker	1127	68.55

Ex-Smoker	197	11.98

Smoker	320	19.46

**Table 3:** Prevalence of obesity and overweight according to general characteristics of the studied population.

Variable	Normal N (%)	Overweight N (%)	Obesity N (%)
*Gender*			

Male	310 (33)	382 (40.7)	246 (26.2)

Female	250 (35.5)	249 (35.4)	205 (29.1)

*Nationality*			

Saudi	410 (34)	468 (38.8)	328 (27.2)

Non Saudi	115 (35.9)	120 (37.5)	85 (26.6)

*Marital Status*			

Married	264 (45.1)	199 (34)	123 (21)

Single	258 (28)	384 (41.6)	281 (30.4)

Divorced	33 (29.5)	42 (37.5)	37 (33)

*Level of Education*			

Primary	301 (35.6)	308 (36.4)	236 (27.9)

Secondary	130 (29.8)	193 (44.3)	113 (25.9)

University	120 (36.5)	113 (34.3)	96 (29.2)

*Monthly Income*			

>7000 SAR	265 (40.9)	227 (35)	156 (24.1)

5000-7000 SAR	84 (29.3)	129 (44.9)	74 (25.8)

<5000 SAR	111 (23.7)	198 (42.2)	160 (34.1)

*Hypertension*			

Yes	62 (24.31)	83 (32.54)	110 (43.13)

No	499 (35.09)	539 (37.90)	384 (27.00)

*Smoking Status*			

Non-Smoker	397 (35.25)	370 (32.85)	360 (31.94)

Ex-Smoker	66 (33.50)	43 (21.82)	88 (44.67)

Smoker	151 (47.18)	72 (22.5)	97 (30.31)

Overweight was defined as BMI equal to or greater than 25. Obesity was defined as BMI equal to or greater than 30.

## Data Availability

The data used to support the findings of this study are restricted by the Ethics Board, Scientific Research and Community Service Committee, Faculty of Medicine, Najran University, Kingdom of Saudi Arabia, in order to protect patient privacy. Data are available from Dr. Awad Mohammed Al-Qahtani (contact details: mobile: 00966530540450; email: awadresearch17@gmail.com) for researchers who meet the criteria for access to confidential data.
