# Corrigendum to “Prevalence and Predictors of Obesity and Overweight among Adults Visiting Primary Care Settings in the Southwestern Region, Saudi Arabia”

**DOI:** 10.1155/2020/3796923

**Published:** 2020-10-27

**Authors:** Awad Mohammed Al-Qahtani

**Affiliations:** Department of Family and Community Medicine, College of Medicine, Najran University, Najran, Saudi Arabia

In the article titled “Prevalence and Predictors of Obesity and Overweight among Adults Visiting Primary Care Settings in the Southwestern Region, Saudi Arabia” [1], information was omitted from Acknowledgments in error. Acknowledgments is shown below.
